# Retraction: Progesterone potentiates the growth inhibitory effects of calcitriol in endometrial cancer via suppression of CYP24A1

**DOI:** 10.18632/oncotarget.28904

**Published:** 2026-07-21

**Authors:** Amber A. Bokhari, Laura R. Lee, Dewayne Raboteau, Jane Turbov, Isabel V. Rodriguez, John Wesley Pike, Chad A. Hamilton, George Larry Maxwell, Gustavo C. Rodriguez, Viqar Syed

**Affiliations:** ^1^Department of Obstetrics and Gynecology, Uniformed Services University of the Health Sciences, Bethesda, MD, USA; ^2^Division of Gynecologic Oncology, North Shore University Health System, University of Chicago, Evanston, IL, USA; ^3^Department of Biochemistry, University of Wisconsin, Madison, WI, USA; ^4^Division of Gynecologic Oncology, and Gynecologic Cancer Translational Research Center of Excellence, Walter Reed National Military Medical Center, Bethesda, MD, USA; ^5^John P. Murtha Cancer Center at Water Reed National Military Medical Center, Bethesda, MD, USA; ^6^Department of Obstetrics and Gynecology and Women’s Health Integrated Research Center, Inova Fairfax Hospital, Falls Church, VA, USA; ^7^Department of Molecular and Cell Biology, Uniformed Services University of the Health Sciences, Bethesda, MD, USA

**This article has been retracted:** Oncotarget has completed its investigation and found multiple internal duplications of western blot (WB) images and also external duplication. Specifically, in Figure 4 “Immunoblot analysis of CYP24A1 protein levels in HEC-1B, Ishikawa, and RL-95 cells after treatment with vehicle (control), progesterone (PROG), medroxyprogesterone (MPA), norgestrel (NORG), or norethindrone (NOR) alone or in combination with calcitriol for 8, 24, 72 or 120 h.”, we found duplicated β-actin images for RL-95 cells which belonged to different treatment groups at different treatment times (8 and 72 hours, and 24 and 120 hours). Additionally, CYP24A1 images for different cell lines (Ishikawa and RL-95) and treatments (NORG vs NOR) were duplicated. Similarly, in Figure 5 “Effects of actinomycin D and cycloheximide on calcitriol-induced CYP24A1 splicing.”, RT-PCR images in panel 5B, pertaining to HEC-1B and Ishikawa cells treated with or without Cycloheximide at various time points, exhibited multiple duplicated or spliced regions. Furthermore, an external duplications were identified in Figure 4, where WB image for CYP24A1 in Ishikawa cells treated for 120 hours in the MPA group was previously published as a p27kip1 WB image in Figure 4E of an unrelated 2015 paper [1] and CYP24A1 blot in HEC-1B cells treated for 72 h in the NORG group was found in Figure 3D of 2016 unrelated paper [2] as blot for p-AMPK in MDA-MB-231 cells. And more were identified in Figure 5A where RT-PCR 18S images in Actinimycin treated groups for HEC-1B cells (2 h) and Ishikava cells (30 min) are duplicates of RT-PCR images for GAPDH control in Figures 1C and 2A of unrelated 2012 article, respectively [3]. Furthermore, in Figure 8B an image for β-actin in HEC-1B cells in a scrambled siRNA-transfected group was previously published as a GAPDH WB image in Figure 4C of an unrelated 2011 paper [4] and MMP-2 WB was found as Cleaved PARP blot in Figure 4D of unrelated article [5].

Given these findings, the Editors have lost confidence in the results and conclusions presented in this article, leading to the decision to retract it. The authors have been notified of this decision. To date, only Dr. Laura Lee has responded and expressed agreement with the retraction. The remaining authors have either not replied or could not be reached.

Original article: Oncotarget. 2016; 7:77576–77590. 77576-77590. https://doi.org/10.18632/oncotarget.12725
